# Six-Fraction Ultra-Hypofractionated Radiotherapy for Tumours Adjacent to or Involving the Brachial Plexus: A Retrospective Cohort Study

**DOI:** 10.3390/cancers18193189

**Published:** 2026-10-02

**Authors:** Maximilian Sturz, Gabriela Studer, Christoph Glanzmann, Sergejs Unterkirhers, Philippe Logaritsch, Olga Unterkirhere

**Affiliations:** 1 Department of Radiation Oncology, University Teaching Hospital Luzern, Spitalstrasse, 6004 Luzern, Switzerland; 2 Medical Physics, Radiotherapy Hirslanden, Witellikerstrasse 40, 8032 Zurich, Switzerland

**Keywords:** palliative radiotherapy, brachial plexus, tumour-related plexopathy, ultra-hypofractionation

## Abstract

**Background/Objectives:** Tumours abutting or involving the brachial plexus can cause pain and neurological deficits. We evaluated six-fraction ultra-hypofractionated radiotherapy with a simultaneous integrated boost (uhRT-SIB) in a predominantly palliative cohort. **Methods:** We retrospectively analysed consecutive patients treated from April 2022 to June 2026, with a data cut-off of 7 July 2026. Image-guided VMAT delivered 33 Gy and 24 Gy to the high- and lower-dose planning target volumes, respectively. Outcomes included the best documented CTCAE-based symptom response, study-defined volumetric response (>30% GTV reduction), freedom from local progression (FFLP), overall survival (OS), and neurological findings in routine clinical records. **Results:** Forty-four patients with 45 lesions were treated; 29/45 lesions (64.4%) contacted the plexus. The median observed follow-up was 7.8 months (range 0.6–40.6). Symptoms improved by at least one grade in 16/19 evaluable symptomatic patients (84.2%, 95% CI 62.4–94.5%). The median times to first documented benefit and best symptom grade among responders were 9 and 23 days. The first-scan volumetric response occurred in 24/30 evaluable lesions (80.0%, 95% CI 62.7–90.5%). Six- and 12-month FFLP were 84.7% (95% CI 63.9–94.0%) and 73.7% (95% CI 49.7–87.6%), respectively. The median OS was 9.6 months. No clinical diagnosis of radiation-induced brachial plexopathy was documented; neurological assessment was not standardised. **Conclusions:** In this predominantly palliative cohort, six-fraction uhRT-SIB was associated with symptom improvement in 84.2% of evaluable symptomatic patients, study-defined volumetric response in 80.0% of evaluable lesions, and 12-month FFLP of 73.7%. No clinical diagnosis of radiation-induced brachial plexopathy was documented during routine follow-up, although the assessment of late neurological toxicity was limited.

## Simple Summary

Malignant tumours close to or involving the brachial plexus can cause severe pain, sensory loss, and weakness. Delivering an effective radiation dose is challenging because the plexus is also vulnerable to radiation injury. We reviewed 44 patients with 45 lesions treated with a six-fraction, image-guided radiotherapy regimen using a simultaneous integrated boost. Symptoms improved in 16 of 19 evaluable symptomatic patients, and tumour volume decreased by more than 30% in 24 of 30 evaluable lesions. Freedom from local progression at 12 months was 73.7%. No clinical diagnosis of radiation-induced brachial plexopathy was documented, although neurological follow-up was not standardised. The observed symptom improvement and local control support further evaluation of this short-course approach for selected patients requiring palliation.

## 1. Introduction

Malignant involvement of the brachial plexus can cause severe neuropathic pain, sensory loss, motor weakness, and functional impairment, particularly in patients with advanced lung or breast cancer [1,2]. Radiotherapy may relieve tumour-related symptoms and provide local control, but treatment planning is challenging when gross disease abuts, displaces, or encases the plexus.

This clinical need must be balanced against radiation-induced brachial plexopathy (RIBP), a potentially irreversible late toxicity that can be difficult to distinguish from tumour progression or metastatic plexopathy [2,3]. In conventionally fractionated series, a systematic review and meta-analysis supported maximum-dose constraints of approximately 60–66 Gy and found increasing risk with increasing plexus Dmax [4]. Prospective symptom screening in head-and-neck cancer also demonstrated dose-volume effects, particularly when more than 10% of the ipsilateral plexus received at least 70 Gy [5]. Older breast cancer data further indicate that large fraction sizes and treatment-related hot spots can substantially increase risk [6,7].

Evidence for stereotactic and markedly hypofractionated radiotherapy is less uniform. Published constraints and pooled models differ according to fractionation, contouring method, dose metric, and toxicity ascertainment [8,9,10,11,12,13,14,15]. Forquer et al. reported a higher RIBP risk above 26 Gy in three to four fractions, whereas later pooled models estimated an approximately 10% risk at Dmax values of 32–34 Gy in three fractions and 40–43 Gy in five fractions [9,11,12,13]. A more recent individual-patient pooled analysis estimated corresponding 10% risk levels of 34.2 Gy in three fractions and 42.7 Gy in five fractions [15]. Anatomical contouring is also important because vessel-based surrogates may substantially misestimate the dose to the plexus [14].

Most contemporary evidence concerns an initially neurologically intact plexus adjacent to apical lung tumours and therefore addresses tolerance rather than palliation of pre-existing tumour-related plexopathy. Evidence on modern image-guided short-course radiotherapy for tumours directly abutting or encasing the plexus remains sparse. For patients treated with palliative intent, protracted daily radiotherapy courses may be burdensome, supporting evaluation of shorter regimens that can provide symptom relief and local control. We evaluated a consecutive cohort treated with a uniform six-fraction uhRT-SIB regimen, combining clinical symptom outcomes with serial volumetry, anatomical plexus dosimetry, and exploratory tumour–plexus geometry. Outcomes included the best documented CTCAE-based symptom response, study-defined volumetric tumour response, FFLP, OS, and neurological findings recorded during routine follow-up.

## 2. Materials and Methods

### 2.1. Study Cohort and Treatment

We retrospectively analysed 44 consecutive patients with 45 lesions adjacent to or involving the brachial plexus treated at our institution between April 2022 and June 2026. The cohort was predominantly treated with palliative intent. The data cut-off was 7 July 2026. One patient contributed two anatomically distinct lesions. Patient-level endpoints, including demographics, symptoms, and OS, were counted once; lesion-level dosimetry, radiographic response, and local progression were analysed per lesion.

All lesions received image-guided uhRT-SIB, prescribing 33 Gy to a high-dose planning target volume (PTV33) encompassing gross disease or, in one postoperative case, the high-risk surgical-bed target and 24 Gy to a lower-dose planning target volume (PTV24), delivered in six fractions. PTV margins were 5 mm for PTV24 and 0–5 mm for PTV33. The PTV33 margin was selected at the discretion of the treating radiation oncologist. Margin reduction was not systematically applied to lesions in direct contact with the plexus. Treatments were delivered on a Varian TrueBeam linear accelerator using volumetric modulated arc therapy (VMAT) with daily cone-beam CT image guidance. Concurrent systemic therapy was administered when clinically indicated according to multidisciplinary tumour board recommendations.

### 2.2. Contouring and Tumour–Plexus Geometry

The brachial plexus was initially contoured on planning CT scans using the commercial deep learning-based autocontouring platform Limbus Contour (Limbus AI), with every contour subsequently reviewed and edited by experienced radiation oncologists. Gross tumour volumes (GTVs) were defined using planning and diagnostic imaging. The spatial relationship between the GTV and brachial plexus was classified as no contact (no direct interface), tangential contact (focal abutment without circumferential extension), semicircular contact (partial circumferential extension around the plexus), or circular contact (near-complete or complete encasement) (Figure 1).

### 2.3. Follow-Up and CTCAE-Based Symptom Assessment

Follow-up was clinically driven rather than protocol mandated. Assessments were performed during routine care in Radiation Oncology, Medical Oncology, or other involved specialties. Outcomes were derived from the electronic medical record, including clinic notes, patient-reported symptom documentation, radiology reports, and treatment-planning data. Symptom and imaging assessments were not scheduled at predefined study time points.

Brachial plexus-related symptoms, including pain, sensory disturbance, and motor weakness, were retrospectively graded according to the Common Terminology Criteria for Adverse Events (CTCAE) v5.0 framework [16]. For symptom assessment, G0 denoted documented absence or resolution of the relevant symptom; observed grades ranged from G0 to G3. The overall symptom grade was the highest documented grade across relevant symptom domains. Symptom response was defined as a reduction in at least one grade from the baseline to the best documented assessment during or after radiotherapy. The time to first documented benefit and the time to the first assessment recording the best symptom grade were extracted separately, both measured from the first RT fraction.

For patients with baseline pain, changes in analgesics, co-analgesics, and corticosteroids between the baseline and the assessment recording the best symptom grade were reconstructed where possible. Medication exposure was classified qualitatively as reduced, unchanged, increased, or not reliably reconstructable.

Possible radiation-induced brachial plexopathy was assessed from the available routine oncological records. There was no protocol for scheduled specialist neurological examinations or systematic neurological screening. Documentation was largely prompted by reported symptoms.

### 2.4. Volumetric Response and Time-to-Event Endpoints

Tumour response at the first evaluable post-treatment scan was assessed by three-dimensional GTV measurements on follow-up CT, referenced to the recorded baseline GTV. All 95 recorded follow-up volume measurements across 31 lesions were CT-based; none used MRI. Two patients underwent additional MRI examinations that were not used for the volumetric measurements. Baseline GTVs were delineated for treatment planning using planning CT and available diagnostic imaging. A study-specific volumetric scheme was used: near-complete volumetric response (nCR), a >90% reduction; partial response (PR), a >30% to 90% reduction; stable disease (SD), a ≤30% reduction to a ≤20% increase; and progressive disease (PD), a >20% increase. The study-defined volumetric response rate (VRR) comprised nCR plus PR. These labels describe percentage changes in three-dimensional tumour volume and are not RECIST 1.1 categories [17]. In particular, nCR allowed residual measurable tissue, and a >30% volume reduction is not equivalent to a ≥30% decrease in the sum of target-lesion diameters. One postoperative lesion had no measurable residual baseline GTV and was excluded from percentage-based response assessment.

PD at the first post-treatment imaging assessment was defined as an increase of more than 20% relative to the baseline GTV. For lesions without progression at the first assessment, subsequent local progression was defined as an increase of more than 20% relative to the smallest previously documented post-treatment tumour volume (post-treatment nadir). For the postoperative lesion without a measurable residual baseline GTV, local progression was defined as a newly measurable in-field recurrence. The date of local progression was the first imaging date fulfilling the respective criterion. FFLP was calculated from the first fraction of radiotherapy to documented local progression, with censoring at the last available local imaging assessment; death was not counted as an event. OS was calculated from the first fraction of radiotherapy to death from any cause or last follow-up. Progression referenced exclusively to the baseline GTV was examined as a sensitivity analysis. In a separate RECIST-inspired volumetric robustness analysis, CR required complete disappearance of the treated-lesion GTV, PR required a ≥65.7% volume reduction from the baseline, and PD required a ≥72.8% volume increase from the smallest previously recorded volume, including the baseline. Best response was assessed up to the first RECIST-inspired PD assessment.

### 2.5. Dosimetry and Statistical Analysis

Dosimetric parameters were extracted from dose-volume histograms, including D0.03cc of the brachial plexus, with the absolute plexus volume receiving ≥30 Gy (V30, cm^3^), and overlap volumes between the plexus and PTV33 and PTV24. D0.03cc was used as a near-maximum dose metric and is not identical to the absolute point maximum reported in several historical SBRT series.

Continuous variables were summarised using the mean, median, interquartile range, and range as appropriate. Proportions are reported with 95% Wilson score confidence intervals. Associations between continuous variables were assessed using Spearman rank correlation. Continuous variables were compared between two groups using the Mann–Whitney U test, and categorical 2 × 2 comparisons used two-sided Fisher’s exact tests. Kaplan–Meier estimates were reported with complementary log–log confidence intervals, and geometry subgroup comparisons used the log-rank test. In the 31-lesion imaging-evaluable cohort, the cumulative incidence of documented local progression was estimated using the Aalen–Johansen approach, with death without documented local progression treated as a competing event and other observations censored at the last available local imaging assessment. Median follow-up was estimated using reverse Kaplan–Meier, with deaths censored and last contacts in surviving patients treated as events.

Exploratory analyses compared symptom response at the patient level and first-scan volumetric response at the lesion level according to recorded concurrent systemic therapy status. Baseline characteristics and OS were also described for patients with versus without post-treatment imaging. Because imaging availability was determined after treatment and depended partly on survival, this comparison was descriptive, and no causal or adjusted treatment effect was estimated. Analyses used available observations without imputing missing responses. All analyses were exploratory and unadjusted for multiple testing; *p* values were interpreted alongside group sizes and confidence intervals.

Data handling, statistical analyses, and graphical presentation were performed in Python 3.10 using pandas 1.5, NumPy 1.23, SciPy 1.9, and custom functions that were independently cross-checked against the raw time-to-event data. Analyses were exploratory; no adjustment for multiple testing was performed. Two-sided *p* values < 0.05 were considered statistically significant.

The project was approved by the Ethics Committee of Northwest and Central Switzerland as a reuse-of-data study (EKNZ Project ID 2026-00488). Data were coded and handled in accordance with the ethics approval and applicable Swiss legislation.

## 3. Results

### 3.1. Cohort and Follow-Up Availability

The cohort comprised 44 patients and 45 treated lesions. A best documented symptom grade during or after radiotherapy was available for 19/21 patients with baseline symptoms (90.5%). Follow-up imaging was available for 31/45 lesions (68.9%) in 31 patients; 13 patients with 14 lesions had no recorded post-treatment imaging. Percentage-based volumetric response was evaluable in 30/45 lesions (66.7%), after exclusion of the postoperative lesion without a measurable baseline GTV. The median observed time from the first RT fraction to death or last contact was 7.8 months (IQR, 2.7–18.3; range, 0.6–40.6). The median follow-up estimated using reverse Kaplan–Meier was 31.5 months. Patient- and lesion-level characteristics are summarised separately in Table 1.

Post-treatment imaging was available for 31 patients and unavailable for 13. Baseline plexus symptoms were present in 15/31 patients (48.4%) versus 6/13 (46.2%), and concurrent systemic therapy was recorded in 13/31 (41.9%) versus 4/13 (30.8%). The median OS was 14.2 months in patients with imaging and 2.8 months in those without imaging. Nine of the 13 patients without imaging had died, while four were alive at their last recorded contact.

### 3.2. Brachial Plexus Involvement and Dosimetry

Overall, 29/45 lesions (64.4%) demonstrated direct anatomical contact with the brachial plexus (tangential/semicircular/circular), whereas 16/45 (35.6%) had no contact. In the overall cohort (*n* = 45), brachial plexus D0.03cc had a mean/median of 31.5/32.4 Gy (range, 24.4–34.5), and high-dose PTV33–plexus overlap had a mean/median of 1.46/0.26 cm^3^ (range, 0.00–25.52). Among lesions with direct plexus contact (*n* = 29), D0.03cc had a mean/median of 32.6/33.1 Gy (range, 24.9–34.5), and PTV33–plexus overlap had a mean/median of 2.21/0.59 cm^3^ (range, 0.00–25.52).

### 3.3. CTCAE-Based Symptom Response

At baseline, 21/44 patients (47.7%) had plexus-related symptoms: pain only in 11/21 (52.4%), isolated neurological symptoms in 1/21 (4.8%), and combined pain and sensory and/or motor symptoms in 9/21 (42.9%). A best documented symptom grade was available in 19/21 symptomatic patients (90.5%). Overall, 16/19 (84.2%, 95% CI 62.4–94.5%) improved by at least one CTCAE-based symptom grade, 3/19 (15.8%) had no grade reduction, and none of these 19 patients had a higher best recorded grade than at baseline. Two symptomatic patients (9.5%) were not evaluable. Among the 16 responders, the median time from the first RT fraction to first documented benefit was 9.0 days (IQR, 7.0–37.3; range, 6–185). The first assessment recording the best symptom grade occurred at a median of 15.0 days among all 19 evaluable patients (IQR, 7.0–56.5; range, 6–263) and 23.0 days among the 16 responders (IQR, 7.0–62.5; range, 6–263). Individual symptom-grade transitions and times to first documented benefit are shown in Figure 2.

Among 10 evaluable patients with isolated pain, 6/10 (60.0%) achieved complete resolution to G0, 3/10 (30.0%) improved to G1, and 1/10 (10.0%) remained stable at G1. The patient with isolated sensory symptoms improved to G0. Among eight evaluable patients with combined pain and neurological deficits, 2/8 (25.0%) achieved complete resolution, 4/8 (50.0%) improved to G1, and 2/8 (25.0%) had persistent G2–G3 symptoms. These symptom-response observations were distinct from the assessment of possible radiation-induced plexopathy.

Symptom improvement occurred in 6/8 evaluable patients receiving concurrent systemic therapy (75.0%, 95% CI 40.9–92.9%) and 10/11 without concurrent systemic therapy (90.9%, 95% CI 62.3–98.4%; Fisher *p* = 0.546).

Among 18 evaluable patients with pain at baseline, medication was reduced in 2/18 (11.1%), unchanged in 6/18 (33.3%), and increased in 3/18 (16.7%); the course could not be reliably reconstructed in 7/18 (38.9%). Overall symptom-grade improvement occurred in 2/2, 5/6, 2/3, and 6/7 patients in these respective categories. Exact doses were not consistently available, and these counts do not represent an analgesic-adjusted pain-response analysis.

### 3.4. Volumetric Response and Local Progression

Follow-up CT was available for 31/45 lesions (68.9%). Among the 30 lesions with evaluable percentage-based volumetric measurements, the first post-treatment assessment occurred after a median of 64.0 days from RT completion (IQR 35.3–100.0; range 7–251). The interval from RT completion to the first volumetric assessment was moderately correlated with the measured percentage volume reduction (Spearman ρ = 0.50, *p* = 0.005). One postoperative lesion had no measurable baseline GTV and was excluded from these response analyses.

At the first evaluable post-treatment scan, 10/30 lesions (33.3%) had nCR, 14/30 (46.7%) PR, 4/30 (13.3%) SD, and 2/30 (6.7%) PD, yielding a study-defined VRR of 24/30 (80.0%, 95% CI 62.7–90.5%). Across all 30 evaluable lesions, the median GTV reduction from the baseline was 70.1% (IQR 45.7–93.6%). In the stricter RECIST-inspired robustness analysis, the best complete or partial response was achieved in 18/30 lesions (60.0%, 95% CI 42.3–75.4%).

The first-scan VRR was 12/13 lesions with concurrent systemic therapy (92.3%, 95% CI 66.7–98.6%) and 12/17 without concurrent systemic therapy (70.6%, 95% CI 46.9–86.7%; Fisher *p* = 0.196). The wide intervals limit interpretation of the apparent difference.

The FFLP analysis included 31 lesions with post-treatment imaging. Using baseline-referenced PD at the first post-treatment assessment and nadir-referenced progression thereafter, FFLP was 84.7% (95% CI 63.9–94.0%) at 6 months and 73.7% (95% CI 49.7–87.6%) at 12 months (Figure 3). Eight local progression events were observed: two at the first assessment relative to the baseline and six during subsequent follow-up relative to the post-treatment nadir. Within this same 31-lesion imaging-evaluable cohort, the 12-month cumulative incidence of documented local progression was 20.1%, with death without documented local progression treated as a competing event.

### 3.5. Overall Survival and Neurological Findings

At data cut-off, 17/44 patients (38.6%) were alive. The median OS from the first fraction of radiotherapy was 9.6 months. The estimated OS was 59.0% (95% CI 42.6–72.2%) at 6 months and 45.5% (95% CI 29.6–60.0%) at 12 months (Figure 4).

No clinical diagnosis of radiation-induced brachial plexopathy was documented in the available routine oncological follow-up records. Neurological reporting was symptom-driven and was not supported by scheduled specialist examinations or standardised screening. Across the full cohort, 24 patients had an overall observation period of at least 6 months, 16 at least 12 months, and nine at least 24 months from the first RT fraction to death or last contact. These durations describe general clinical observations, not repeated neurological assessments. Consequently, the records did not support a reliable estimate of the incidence or latency of late neurological toxicity.

### 3.6. Exploratory Associations with Tumour–Plexus Geometry

Study-defined early volumetric response was observed across all contact patterns: the VRR was 2/4 (50.0%) for circular lesions, 10/12 (83.3%) for tangential lesions, 2/2 (100%) for semicircular lesions, and 10/12 (83.3%) for lesions without contact. These estimates were based on small subgroups.

Circular encasement showed numerically inferior FFLP in the imaging follow-up cohort. Local progression occurred in 2/4 circular lesions (50.0%) and 6/27 non-circular lesions (22.2%; Fisher *p* = 0.268). Twelve-month FFLP was 50.0% versus 78.1%, respectively (log-rank *p* = 0.151; Figure 5). Because only four circular lesions had imaging follow-up and only eight local events occurred, this finding should be regarded as hypothesis-generating.

Circular lesions were substantially larger than non-circular lesions (median baseline GTV of 120.6 vs. 25.3 cm^3^; Mann–Whitney *p* = 0.004), indicating potential confounding between tumour burden and geometry.

## 4. Discussion

In this retrospective, predominantly palliative cohort, six-fraction uhRT-SIB was associated with best documented symptom improvement in 16/19 evaluable symptomatic patients (84.2%), a study-defined first-scan VRR of 24/30 lesions (80.0%), and a 12-month FFLP of 73.7%. In the imaging-evaluable cohort, the 12-month cumulative incidence of documented local progression was 20.1% with death as a competing event. No clinical diagnosis of RIBP was documented in the available routine follow-up records, although neurological ascertainment was limited.

The palliative context is central to interpretation. Treatment aimed to provide local tumour control and, in symptomatic patients, relief of tumour-related plexopathy. A median treatment time of 7 days may be useful when prolonged attendance is burdensome. Patient selection should therefore take account of treatment intent, expected survival, symptom burden, and disease extent, including metastatic status, previous treatments, and available systemic options. The anticipated benefit in symptom relief and local control should be weighed against the burden of treatment and the patient’s priorities. However, 17/44 patients received concurrent systemic therapy, and the small exploratory comparisons showed wide confidence intervals for both symptom and volumetric response. These comparisons cannot isolate the contribution of radiotherapy from systemic treatment. Changes in analgesics, co-analgesics, and corticosteroids provide an additional explanation for symptom improvement: medication was increased in three of 18 evaluable patients with baseline pain, while medication trajectories could not be reconstructed reliably in seven patients. The available data therefore describe a documented symptom-grade improvement rather than an analgesic-adjusted pain response or a treatment-specific causal effect.

The observed symptom improvement is broadly consistent with historical palliative radiotherapy reports despite substantial differences in treatment era, patient selection, dose, technique, systemic therapy, and outcome assessment (Table 2). Nisce and Chu reported pain relief in 35/47 patients with breast cancer-related plexus syndrome [18]. Ampil observed significant pain relief in 17/22 patients and objective tumour response in 6/13 evaluable cases [19]. Van Houtte et al. reported symptomatic relief in 21/31 patients with superior sulcus tumours, with more frequent palliation after doses above 50 Gy [20]. Kamenova et al. reported symptom remission in 19/22 patients treated with radiotherapy and/or systemic therapy [21]. The contribution of the present study is the description of a uniform ultra-hypofractionated, image-guided SIB regimen delivered in six fractions, with anatomical brachial plexus dosimetry, serial tumour volumetry, and documented symptom outcomes. In this predominantly palliative population with limited life expectancy, limiting the number of treatment visits is an important consideration alongside symptom relief and local tumour control. The observed clinical outcomes support the relevance of this short-course approach for selected patients, with fewer treatment visits than more protracted radiotherapy schedules. These retrospective findings do not establish superiority over other palliative regimens or define a new brachial plexus tolerance limit.

Tumour shrinkage and classification of response should be distinguished. The median volume reduction of 70.1% describes the measured change continuously, whereas the VRR of 80.0% depends on the study-specific threshold of a >30% volume reduction at the first evaluable CT. This threshold is less stringent than the volume change corresponding to a 30% diameter reduction for a similarly shaped lesion, and it cannot be interpreted as a RECIST response rate [17]. The stricter RECIST-inspired analysis identified the best complete or partial response in 18/30 lesions (60.0%). It used both different volume thresholds and a different assessment window, assessing the best response across serial scans rather than response at the first scan; it is therefore a complementary description, not a direct validation of the primary VRR. All analysed follow-up volumetry used CT, but assessment times varied from 7 to 251 days after RT completion and longer intervals were associated with greater measured regression. Variation in imaging indication, acquisition, timing, and segmentation remains a source of measurement and ascertainment bias.

The local endpoint also requires precise interpretation. Because death was not counted as an event in the Kaplan–Meier analysis, the estimand is FFLP rather than conventional progression-free survival. Nadir referencing captures regrowth after an initial response but can classify progression while a lesion remains smaller than at baseline. An exclusively baseline-referenced sensitivity analysis consequently yielded a higher 12-month FFLP of 88.9%. The competing-risk estimate provides a complementary description of documented progression in the 31-lesion imaging cohort. Neither approach removes the limitations arising from clinically driven imaging or the exclusion of lesions without imaging follow-up.

The imaging-availability comparison shows why the evaluable response rates require caution. Patients with imaging had a median OS of 14.2 months, compared with 2.8 months among those without imaging. Because a patient generally had to remain alive and undergo a subsequent scan to enter the imaging group, this difference reflects selection and a guarantee-time effect rather than an effect of imaging itself. Early death may explain some missing assessments, but four patients without imaging were alive at their last contact. The 80.0% VRR therefore describes the 30 evaluable lesions and should not be extrapolated to all 45 treated lesions.

Tumour–plexus geometry did not preclude early response. Circular lesions had a VRR of 50.0%, compared with 83.3–100% across the other categories, and showed numerically lower 12-month FFLP. However, circular lesions were substantially larger at baseline than non-circular lesions, so the apparent geometry signal may partly reflect tumour burden. This observation remains highly unstable: only four circular lesions had imaging follow-up, two progressed, and only eight local events occurred overall, precluding reliable multivariable modelling. Geometry should therefore be regarded as a hypothesis-generating descriptor rather than a validated prognostic factor.

Plexus exposure was substantial, with a median D0.03cc of 32.4 Gy and a maximum of 34.5 Gy in six fractions. The latter corresponds to approximately 60.4 Gy EQD2 for an alpha/beta ratio of 3 Gy and 66.8 Gy for an alpha/beta ratio of 2 Gy, although linear-quadratic conversion at large fraction sizes and cross-study comparison of D0.03cc with absolute Dmax require caution. Contemporary publications illustrate the uncertainty: consensus tables list five-fraction and eight-fraction point-dose limits of 32.5 and 39.2 Gy for grade ≥3 neuropathy [8], while pooled clinical models provide different grade ≥2 risk estimates [11,12,13,14,15]. The most recent individual-patient analysis estimated a 10% risk at an anatomical-plexus Dmax of 34.2 Gy in three fractions and 42.7 Gy in five fractions [15]. Furthermore, Niu et al. showed that vessel-based surrogates can substantially underestimate anatomical plexus exposure [14]. RIBP in the Bai pooled cohort occurred at a median of 8 months, with a range of 3–54 months [15]. The absence of a documented clinical diagnosis of RIBP in our cohort is an encouraging early observation in a palliative setting. Its interpretation is limited by short observed survival and symptom-driven reporting; mild, subclinical, or late neurological injury could have remained unrecognised. Longer, standardised neurological assessment is needed to characterise the late risk.

This retrospective, single-institutional, uncontrolled study is limited by heterogeneity in tumour type, systemic treatment, baseline symptoms, and follow-up. Symptom grades were reconstructed from routine records without validated prospective patient-reported assessments. Assessment times varied, and longer observation afforded more opportunities to document improvement. Serial GTV segmentation was not prospectively standardised, and interobserver reproducibility of segmentation and the study-specific geometry classification was not assessed. The median observed time from RT to death or last contact was 7.8 months; the reverse Kaplan–Meier estimate of 31.5 months reflects potential follow-up availability and should not be interpreted as the typical duration of neurological surveillance. Few local events precluded reliable multivariable modelling. Prospective studies should include predefined symptom and neurological assessments, validated patient-reported and functional outcomes, medication documentation, standardised imaging intervals, and longer follow-ups.

## 5. Conclusions

In this retrospective, predominantly palliative cohort, six-fraction uhRT-SIB was associated with frequent symptom improvement, substantial volumetric tumour regression, and encouraging local control. These findings support the clinical relevance of this ultra-hypofractionated approach for selected patients with limited life expectancy, for whom symptom relief, local tumour control, and limiting treatment visits are important priorities. No clinical diagnosis of RIBP was documented during routine follow-up, although incomplete, non-standardised neurological assessment and limited survival restrict conclusions regarding late neurological toxicity.

## Figures and Tables

**Figure 1:**
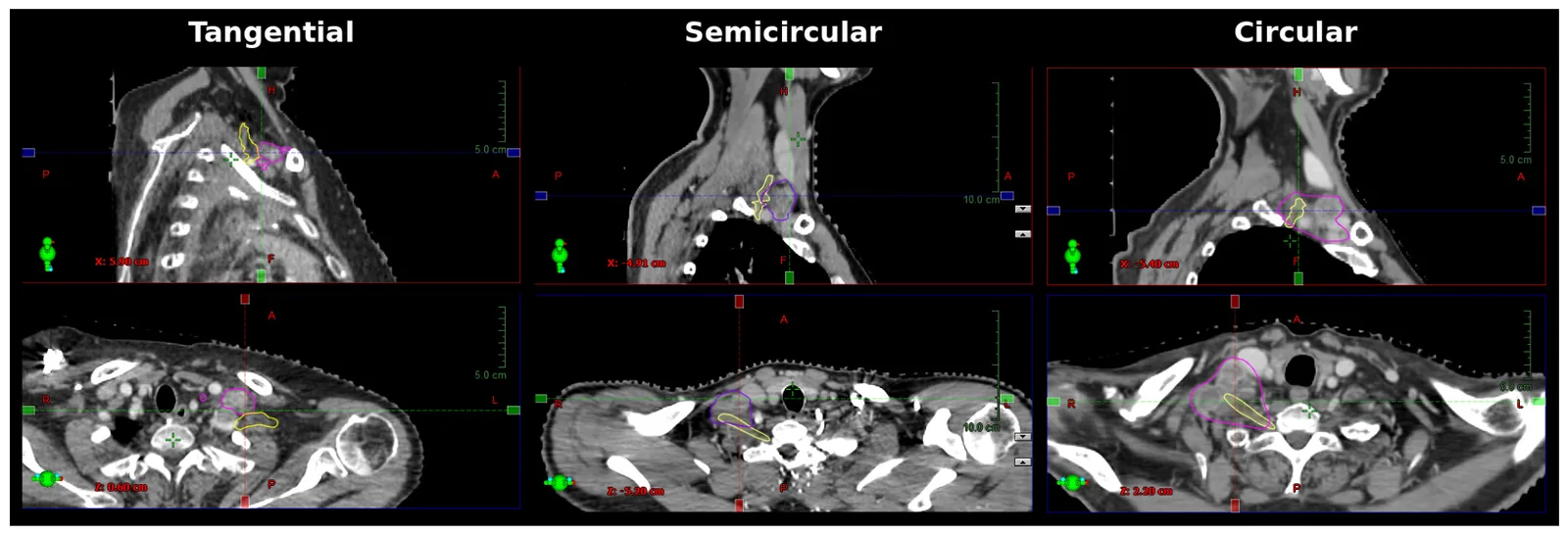
Representative planning CT images illustrating the study-specific tumour–brachial plexus contact patterns: tangential contact, semicircular contact, and circular encasement. Multiplanar reformatted images are displayed in the upper row and axial images in the lower row. The brachial plexus contour is shown in yellow and the tumour contour in magenta/purple. Green markings are treatment-planning display overlays; A, P, R, and L indicate anterior, posterior, right, and left, respectively.

**Figure 2:**
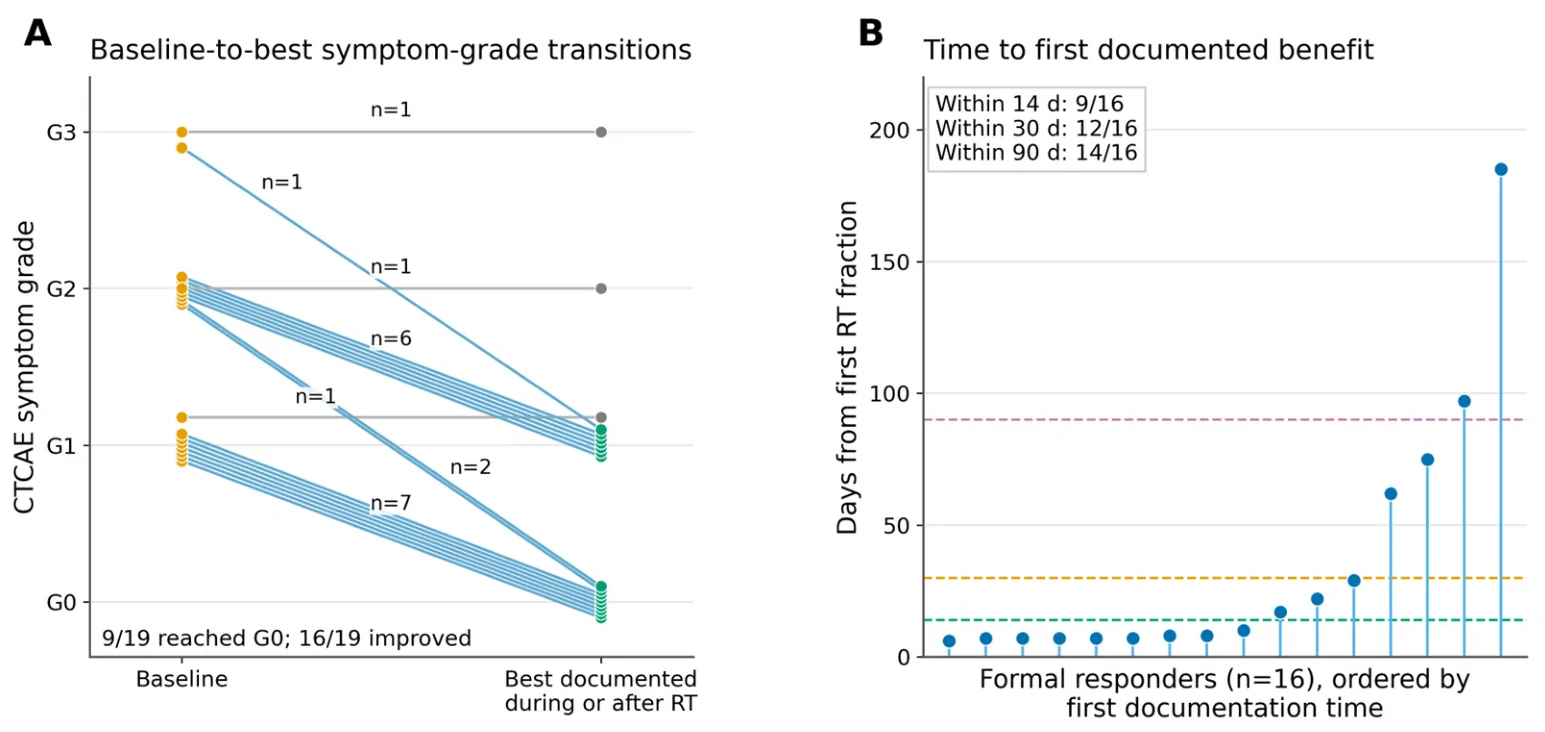
Patient-level CTCAE-based symptom response and time to first documented benefit. (**A**) Transitions from the baseline to the best documented symptom grade during or after radiotherapy among 19 evaluable symptomatic patients. Each line represents one patient. Sixteen patients improved, including nine who achieved complete symptom resolution (G0); three remained unchanged. Baseline grades are shown in orange; improved and unchanged follow-up grades are shown in green and grey, respectively. (**B**) The time from the first radiotherapy fraction to first documented benefit among the 16 responders, ordered by documentation time. Response was defined as an improvement in at least one CTCAE grade; dashed horizontal lines indicate 14, 30, and 90 days.

**Figure 3:**
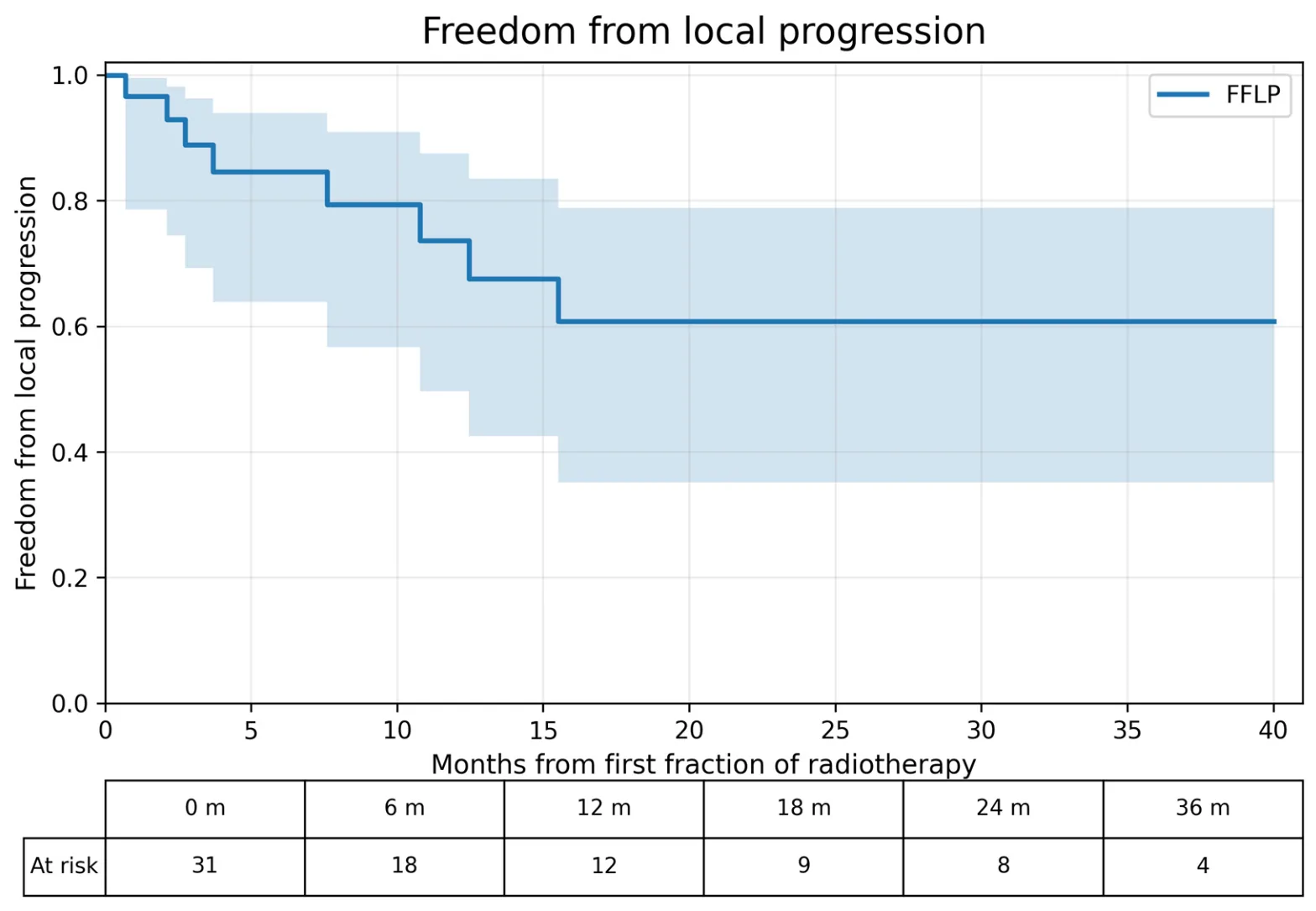
Freedom from local progression among 31 lesions with post-treatment imaging. PD at the first post-treatment assessment was evaluated relative to the baseline GTV, whereas subsequent local progression was defined as an increase of more than 20% relative to the post-treatment nadir. Eight local progression events were observed. Lesions were censored at the last available local imaging assessment. Shading indicates the 95% confidence interval.

**Figure 4:**
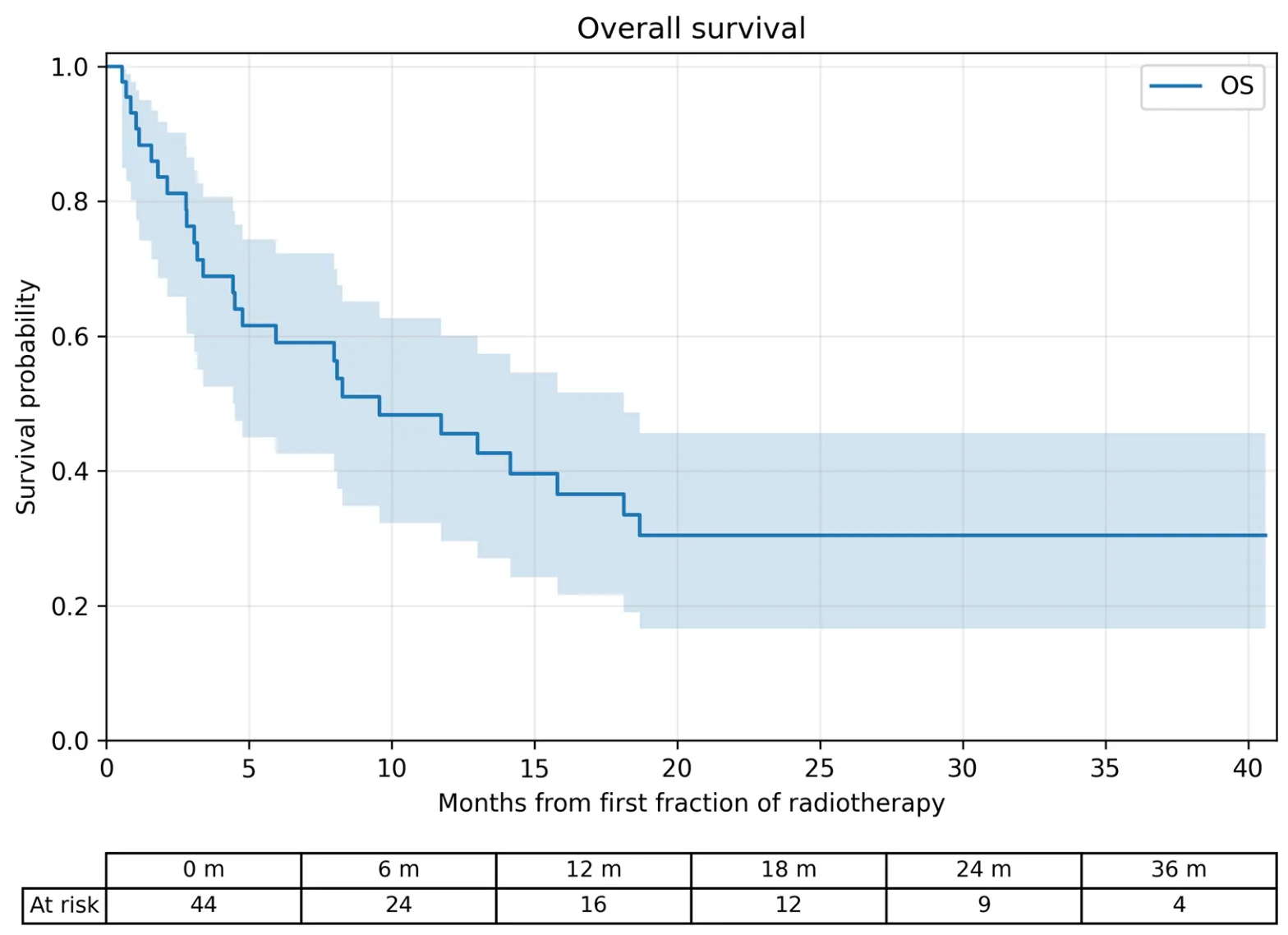
Overall survival from the first fraction of radiotherapy. Shading indicates the 95% confidence interval.

**Figure 5:**
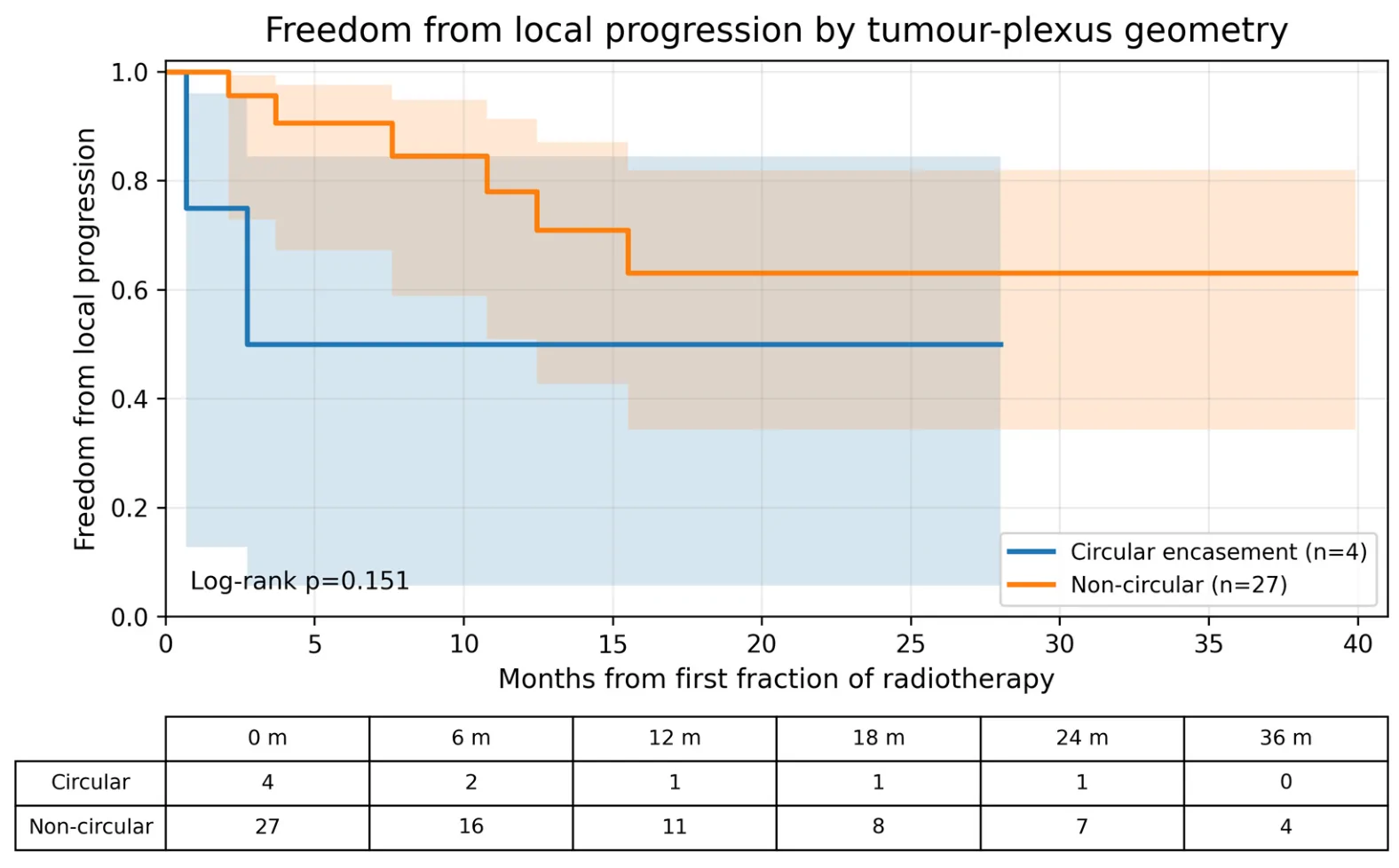
Freedom from local progression stratified by circular versus non-circular tumour–brachial plexus involvement. This exploratory comparison included four circular and 27 non-circular lesions; the difference was not statistically significant (log-rank *p* = 0.151). Shading indicates 95% confidence intervals.

**Table 1:** Baseline patient, tumour, treatment, and dosimetric characteristics.

Characteristic	*N* (%)/Median [IQR] (Min-Max)	Evaluable *n*
**Patient-level characteristics (*n* = 44)**		
Sex, *n* (%)		44
• Female	27 (61.4%)	44
• Male	17 (38.6%)	44
Age at RT (years)	69.7 [61.0–79.5] (41.2–92.4)	44
Primary tumour site, *n* (%)		44
• Lung cancer	15 (34.1%)	44
• Breast cancer	14 (31.8%)	44
• Gastric cancer	2 (4.5%)	44
• Mesothelioma	2 (4.5%)	44
• Other malignancies	11 (25.0%)	44
Classification of the treated lesion, *n* (%)		44
• Primary tumour	4 (9.1%)	44
• Metastatic lesion	40 (90.9%)	44
Baseline plexus-related symptom grade (CTCAE v5.0), *n* (%)		44
• G0	23 (52.3%)	44
• G1	9 (20.5%)	44
• G2	10 (22.7%)	44
• G3	2 (4.5%)	44
Symptom pattern among symptomatic patients (*n* = 21), *n* (%)		21
• Pain only	11 (52.4%)	21
• Neurological symptoms only	1 (4.8%)	21
• Pain plus neurological symptoms	9 (42.9%)	21
Concurrent systemic therapy, *n* (%)		44
• None	27 (61.4%)	44
• Immunotherapy	9 (20.5%)	44
• Chemotherapy	5 (11.4%)	44
• Targeted therapy	2 (4.5%)	44
• Chemo-immunotherapy	1 (2.3%)	44
Observed follow-up from first RT fraction (months)	7.8 [2.7–18.3] (0.6–40.6)	44
Follow-up estimated by reverse Kaplan–Meier, median (months)	31.5	44
**Lesion-level and treatment characteristics (*n* = 45)**		
Overall treatment time (days)	7 [7–7] (7–13)	45
Tumour–plexus involvement pattern, *n* (%)		45
• No contact	16 (35.6%)	45
• Tangential contact	14 (31.1%)	45
• Semicircular contact	6 (13.3%)	45
• Circular encasement	9 (20.0%)	45
Affected aspect of the brachial plexus, *n* (%)		45
• Upper plexus	23 (51.1%)	45
• Lower plexus	16 (35.6%)	45
• Total plexus	6 (13.3%)	45
Direct tumour–plexus contact, *n* (%)		45
• No	16 (35.6%)	45
• Yes	29 (64.4%)	45
Baseline GTV (cm^3^)	31.7 [18.9–66.9] (0.0–381.6)	45
High-dose PTV33 (cm^3^)	68.5 [36.3–109.5] (9.3–458.8)	45
Lower-dose PTV24 (cm^3^)	261.2 [158.7–387.8] (52.5–913.9)	45
Brachial plexus D0.03cc (Gy)	32.4 [31.3–33.3] (24.4–34.5)	45
Brachial plexus V30, absolute volume ≥30 Gy (cm^3^)	0.67 [0.12–2.17] (0.00–27.38)	45
PTV33–plexus overlap (cm^3^)	0.26 [0.00–1.07] (0.00–25.52)	45
PTV24–plexus overlap (cm^3^)	5.38 [3.53–7.13] (0.00–29.20)	45

Values are presented as *n* (%) or the median [IQR] (min–max), unless otherwise specified. Patient-level variables were evaluated in 44 unique patients and lesion-level variables in 45 treated lesions. Symptom grading used the CTCAE v5.0 framework. Other primary tumour types (*n* = 1 each) were melanoma, ovarian cancer, appendiceal cancer, endometrial stromal sarcoma, tubo-ovarian cancer, non-melanoma skin cancer, bladder cancer, cholangiocarcinoma, colorectal cancer, parotid cancer, and carcinoma of unknown primary. One postoperative lesion had no measurable residual GTV at baseline. Primary versus metastatic classification refers to the treated lesion, not to the overall patient stage.

**Table 2:** Selected clinical and dosimetric evidence relevant to tumour-related or radiation-induced brachial plexopathy.

Study	*N*	Population	Regimen/Exposure	Median FU	Symptom/Tumour Response	Local Control	Reported RIBP	Key Relevance
**Historical palliative clinical data**								
Nisce & Chu 1968 [18]	47	Breast cancer-related brachial plexus syndrome	20–60 Gy, mostly 40–50 Gy; approximately 2 Gy/fx	NA	Pain relief in 35/47 (74.5%); complete sensory/motor recovery of 15%, partial recovery of 32%	NA	NA	Early evidence that conventional RT could palliate tumour-related plexopathy.
Ampil 1985 [19]	23	Mainly superior sulcus lung tumours; recurrent breast cancer in 4	10–58 Gy in 5–31 fx; most 40–50 Gy	NA	Significant pain relief in 17/22; any relief in 20/22; objective tumour response in 6/13	NA	NA	Pain palliation appeared more frequent after doses > 40 Gy, but treatment and assessment were heterogeneous.
Van Houtte 1984 [20]	31	Superior sulcus NSCLC	20–70 Gy over 2–7 weeks	OS 17 mo	Symptomatic relief in 21/31; median duration in approximately 11 mo	Local failure in 14/31	Not reported	Doses > 50 Gy were associated with more frequent palliation and better local control.
Kamenova 2009 [21]	22	Breast cancer with brachial plexus syndrome from locoregional metastases	Initial treatment modality: locoregional RT (*n* = 8) or systemic therapy (*n* = 14)	12 mo	Overall symptom remission in 19/22 (86%); median duration of 8 mo	NA	Not separately reported for this cohort	Both locoregional RT and systemic therapy could provide sustained relief; attribution to one modality is limited.
**Modern tolerance, contouring, and dose-response evidence**								
Yan 2019 [4]	25 studies; 37 cohorts	Breast, head-and-neck, lung, and other cohorts; 4227 plexuses	Predominantly conventional fractionation	Variable	NA	NA	Incidence varied across cohorts	Meta-analysis supported Dmax constraints of 60–66 Gy and estimated an RIBP risk ratio of 1.11 per additional Gy.
Forquer 2009 [9]	36 patients; 37 lesions	Apical T1-3N0 NSCLC	30–72 Gy in 3–4 fx	13 mo	NA	2 y LC of 89%	7/37 (18.9%); onset of 6–23 mo	A maximum dose > 26 Gy was associated with higher RIBP risk; vessels were used as a plexus surrogate.
Sood 2018 [10]	31; 18 high risk	Apical NSCLC	40–60 Gy in 3–5 fx	17 mo	NA	17/18 high-risk tumours controlled	0/31	No events were observed despite heterogeneous exposure, but follow-up and sample size limited inference.
Kapitanova 2021 [11]	89 patients; 93 lesions	Pooled apical lung SBRT cohorts	Predominantly 3–4 fx; heterogeneous schedules	13/30 mo	NA	NA	14/89; 7 G2, 6 G3, 1 G4	Preliminary pooled model estimated approximately 10% RIBP risk at Dmax 26 Gy in 3–4 fx.
Milano 2023 [13]	221 patients; 229 targets	Pooled apical or upper-lobe lung SBRT cohorts	Predominantly 3–5 fx	13–30 mo	NA	NA	18/221	NTCP modelling estimated approximately 10% risk at Dmax 32–34 Gy/3 fx and 40–43 Gy/5 fx.
Niu 2024 [14]	137 patients; 140 plexuses	Targets within 2 cm of the anatomical plexus	30–60 Gy in 3–8 fx	32.1 mo	NA	NA	11/137; median onset of 7 mo	Subclavian-axillary vessel surrogates could substantially underestimate anatomical plexus dose, especially for posterior apical targets.
Bai 2024 [15]	349 patients; 356 lesions	Individual-patient pooled analysis of apical lung SBRT	27–60 Gy in 1–8 fx	6–113 mo	NA	NA	22/349 (6.3%); median onset of 8 mo	Estimated 10% risk at anatomical-plexus Dmax of 20.7 Gy/1 fx, 34.2 Gy/3 fx, and 42.7 Gy/5 fx.
Present cohort	44 patients; 45 lesions	Mixed primaries; predominantly palliative treatment; tumour adjacent to or involving plexus	33 Gy/6 fx high-dose PTV plus 24 Gy/6 fx lower-dose PTV	7.8 mo observed clinical follow-up	Best documented symptom improvement in 16/19 (84.2%); VRR in 24/30 (80.0%)	6/12 mo FFLP of 84.7%/73.7%	No RIBP diagnosis documented; no standardised neurological assessment	Uniform six-fraction SIB with anatomical dosimetry and documented palliation; late neurological risk remains uncertain.

Abbreviations: Dmax = maximum dose; FFLP = freedom from local progression; FU = follow-up; fx = fraction(s); LC = local control; NA = not available; NSCLC = non-small-cell lung cancer; NTCP = normal tissue complication probability; RIBP = radiation-induced brachial plexopathy; RT = radiotherapy; SBRT = stereotactic body radiotherapy; VRR = study-defined volumetric response rate.

## Data Availability

Coded data supporting the findings of this study are available from the corresponding author upon reasonable request, subject to institutional, legal, and ethical restrictions. Patient-level data are not publicly available because of patient confidentiality and data-protection requirements.

## Abbreviations

The following abbreviations are used in this manuscript:

SIB	Simultaneous integrated boost
SBRT	Stereotactic body radiotherapy
RT	Radiotherapy
uhRT	Ultra-hypofractionated radiotherapy
VMAT	Volumetric modulated arc therapy
GTV	Gross tumour volume
PTV	Planning target volume
BP	Brachial plexus
FFLP	Freedom from local progression
OS	Overall survival
VRR	Study-defined volumetric response rate
RIBP	Radiation-induced brachial plexopathy
EQD2	Equivalent dose in 2 Gy fractions
nCR	Near-complete volumetric response
PR	Partial response
SD	Stable disease
PD	Progressive disease
CIF	Cumulative incidence function
NTCP	Normal tissue complication probability
CTCAE	Common Terminology Criteria for Adverse Events
